# Supplementary material for: Machine Learning based histology phenotyping to investigate the epidemiologic and genetic basis of adipocyte morphology and cardiometabolic traits
Source: PLoS Comput Biol. 2020 Aug 14;16(8):e1008044. doi: 10.1371/journal.pcbi.1008044 (PMC7449405; doi:10.1371/journal.pcbi.1008044)
Supplement: Attachment — Submitted filename: Response to reviewers - Glastonbury 2020.docx [file pcbi.1008044.s005.docx]

Reviewer's Responses to Questions

We thank the reviewers for the time and energy they have taken in reading and providing detailed feedback on our manuscript. As a result of the reviewers’ comments here, we believe the manuscript has improved in clarity, content and downstream use for readers. Below, you will find our detailed responses to each comment in blue text.

**Comments to the Authors:**

**Please note here if the review is uploaded as an attachment.**

Reviewer #1: Glastonbury and colleagues present their results from an interesting study that ultimately did not deliver the desired genetic associations, but reflects an innovative approach and gives excellent insights into how more definitive studies should be performed.

Their approach is conceptually straightforward – they use novel analysis software to identify adipocytes in histological images, and then within the adipocyte component of the image the size of the adipocyte is measured. This new tool is called Adipocyte U-Net, and is shown to be computationally efficient. They then use this as a quantitative phenotypic trait and use genotypic information from the same patients to test for genetic associations with adipocyte size. The idea that we can harvest disease-relevant phenotypic information from histological images and study genetic associations is the most intriguing part of this report:

**Author action item:**

Please add a short discussion somewhere whether this general approach (genetic association studies with quantitative data from histological images is completely novel or has some precedent.

We thank the reviewer for their comment and have added the following to the introduction:

*“For the first time to our knowledge, we use these image-derived adipocyte area estimates to test for associations with BMI, WHR adjusted for BMI (WHRadjBMI), and a range of glycemic traits. “*

And the following to the discussion:

*“Whilst previous studies have used unsupervised learning methods to extract unknown cellular phenotypes and then performed GWAS on these latent representations*[*^37^*](https://paperpile.com/c/VCmO4E/ty62)*, this study is the first to our knowledge that ties specific (supervised) machine learned image phenotypes to genetic variants.”*

The rationale for the study is that increased fat underlies increased body mass index (BMI), and that increased fat is due to either the hyperplasia (number of cells) or hypertrophy (size of cells) of adipocytes. Of these, it is only really practical to measure size, as it would be enormously challenging to count adipocytes accurately.

**Author action item:**

Please discuss whether anyone has ever tried to measure adipocyte number, or even how in theory this might be performed.

*There are indeed published methods specifically designed to count adipocytes, for example AdipoCount (*[*https://www.ncbi.nlm.nih.gov/pmc/articles/PMC5826178/*](https://www.ncbi.nlm.nih.gov/pmc/articles/PMC5826178/)*).*

*Another common and published approach would be to calculate the total or depot specific adipocyte number from fat cell volume. This is best done in floating mature adipocytes from collagenase digestion as you always measure the maximum cell diameter (which is not always the case in histology). Assuming a density of adipose tissue of 0.9 and knowing the total fat mass by either weighing the fat pads (mice) or dexa/bia measurements in humans you can calculate the total number of fat cells in the body. This has for example been done by Spalding et al. (2008). However, we agree with Reviewer #1 that adipocyte surface area is a much more interesting measurement.*

Their Adipocyte U-Net approach is validated by a hold-out dataset, relative to other automated segmentation methods, and relative to data measuring single cell suspensions of adipocytes, performing well in each case.

The authors use samples of tissues from different parts of the body, and from four separate studies, finding that there are differences in sizes of adipocytes by anatomical site in the body, larger adipocytes in visceral fat in individuals with increased BMI, smaller adipocytes in visceral fat in women, but also larger adipocytes in samples with longer time in ischaemia.

**Author action item:**

Where it states “Given the different metabolic and physiological roles subcutaneous and visceral adipose tissue depots play…” a citation would be valuable.

*We agree. We were alluding to the body of evidence that suggests visceral fat has stronger association to insulin resistance, type 2 diabetes and cardiovascular disease. In fact, mendelian randomization analyses support visceral fat mass as a causal risk factor for type 2 diabetes and coronary heart disease (Karlsson et al, 2019, Emdin et al 2017,* [*https://doi.org/10.1038/s41591-019-0563-7*](https://doi.org/10.1038/s41591-019-0563-7)*; doi:10.1001/jama.2016.21042). We have added a citation to that section.*

No association is found for adipocyte size and self-reported ethnicity.

**Author action items:**

With genotyping data available, why was ancestry not assigned using genetic information? How variable were these subjects in terms of their ancestry?

The only multi-ancestry cohort analyzed here was the GTEx cohort; the cohort includes samples of European, African and East Asian ancestry and we did infer ancestry directly from the genetic information (please see the Sample QC section on the GTEx samples for further information). All other cohorts analyzed here were cohorts composed solely of European-descent samples. Additionally, **we accounted** for genetic ancestry in our genome-wide association testing by including the top ten principal components for each cohort (please see ‘Genome-wide association testing and meta-analysis’ for more information). Given the very small numbers of non-European samples included in our data, we did not test for association between ancestry and adipocyte size.

The causality statement “While this suggests that BMI might act as the primary modifier of adipocyte size…” is probably too assertive, as adipocyte size is later described to mediate BMI: “mean adipocyte size in visceral fat accounting for, on average, 25% of the variance of BMI”. These statements are a departure from the usual, more neutral descriptions in terms of associations, in which the direction of causality is not assumed.

We respectfully disagree with the reviewer. First, we have used cautious language using “suggest” and “might '' to describe the relationship. Second, variance explained does not imply any causal association. Variance explained can be correlative, which we do not speculate on here. We have avoided the use of the word *causal* throughout the manuscript as we have not conducted any form of causal analysis (Mendelian randomisation, or randomized trial).

The authors find no loci reaching significance for association with adipocyte size, but then focus on two loci believed to influence adipocyte size, KLF14 and FTO, not finding any evidence to support their roles, but expressing certain valuable caveats in reporting their results.

**Author action item:**

Please explain what a “female-specific type 2 diabetes-imprinted locus” is. Uncited and unclear to a reader.

We apologise if this was unclear. *KLF14* exhibits imprinted expression from the maternal allele. The lead SNP at *KLF14* is associated with T2D, but only in women, hence it is a female-specific diabetes locus. For more detail, please see the original *KLF14* manuscript, which we cite (<https://www.ncbi.nlm.nih.gov/pubmed/29632379>).

The major value of this paper is not in terms of novel insights, but in showing a path to a valuable approach to understanding the intermediate cellular phenotypes in human diseases. The Adipocyte U-Net tool is obviously cell type-specific, but it has to be assumed that other deep learning-based methods can be developed for other cell types.

**Author action item:**

Please comment on how relatively easy or difficult it will be to develop comparable approaches that work for cells other than adipocytes. Are adipocytes relatively easy, will any cell types be predicted to be difficult, what sort of approaches would be need to count cell subtype proportions within a tissue or image area.

Whilst it would take some work to adapt this algorithm to work with other cell types, the algorithm is data efficient and using the principles of transfer learning, it could potentially be fine-tuned on fewer than 50 images of a new cell type. Adipocytes represent a rather easy case as Adipose tissue is quite homogenous. Complicated mixtures, such as the placenta (<https://arxiv.org/abs/1804.03270>) are more difficult, but it is still possible to estimate different populations of cell types present in the tissue histology.

As such, this is a valuable and far-sighted report and should be in the public domain. The GitHub links work, demonstrating the commitment of the authors to sharing code and data.

We thank the reviewer for their feedback and acknowledgement of our data sharing. We agree that computational phenotyping from images will be a commonly used and powerful method for tissue based cellular phenotyping and identifying novel genetic associations in the near future. As we value scientific reproducibility and sharing, we particularly thank the reviewer for pointing out the benefit of us publishing both our code and data.

Reviewer #2: Glastonbury et al. developed a deep-learning method called Adipocyte U-Net to rapidly estimate adipocyte areas from large-scale histology images and use them to study the relationship with obesity-related traits. Applying Adipocyte U-Net to four population-scale cohorts they demonstrate that adipocyte area positively correlates with BMI but GWAS meta-analysis shows no genome-wide significant results with N=820.

The text is well written, and the general presentation is very good. Given the amount of data we generate in daily clinical and scientific life and the enormous potential of machine learning models to make use of these data, the method presented here can be very useful to the scientific community.

The only major point I would like to raise is that the tool in the current state cannot be used by other scientists. Unfortunately, there are no instructions on how to implement, install and run Adipocyte U-Net on the github page. Many data sets and images are available but there is no explanatory text provided and at least for me there was no coherent way how these data should be used. The code is available in rather big .npy scripts, which I was not able to open with jupyter notebook. A user-friendly step-by-step manual would be highly advised. I truly believe that deep learning methods will advance science and medicine but if they are difficult to install and use the majority of users will swerve to tools that are not machine learning based just because of convenience.

We appreciate the reviewer’s comments and want to emphasize that we agree with the reviewer on the importance of transparency and methods and data availability in scientific publishing. Deep learning algorithms are specific, requiring specialised hardware (GPUs) to enable the processing of many terabytes of high resolution histology imaging. This manuscript aims to set out a principle of how such deep learning methods could be applied to imaging data and then coupled to common genetic variants to learn about disease biology. We do feel we have attempted to be as transparent as possible, by sharing all code, data, models, and notebooks to recreate plots from the manuscript.

We would like to emphasise we have shared all data that we can by law (trained models, code, images and annotations) However, we do appreciate the concerns of the reviewer and therefore, to aid our readers in using our software for their own image analysis, and to address the concerns of Reviewer #2, we have provided several updates to our github:

1) We have detailed step by step installation instructions and have tested them using Python 3.5, 3.6, 3.7, on a mac and linux machine environment.
2) We have included a tutorial (command line python script) on the first step of how to perform cell classification using the deep CNN InceptionV3.
3) We have included a tutorial (Jupyter notebook) on how to load Adipocyte U-net and use it to segment images that were classified as containing adipocytes and use these segmentations to estimate adipocyte areas.
4) We have made this tutorial notebook compatible with *mybinder* meaning users can demo the software without having to install it as it is run on a server in a Dockerised container.

As this is open source software, we will continue to develop it and make it as accessible as possible.

Here is a list of comments to further improve this work:

Please add 1-2 sentences in the introduction that list already available image processing tools that are suitable to phenotype adipocytes. A quick search showed AdipoCount (Zhi et al., 2018) and a newer Version of CellProfiler (McQuin et al., 2018), there might be more.

Thank you for the suggestion. We have expanded the introduction with additional citations for methods that perform adipocyte cell quantification.

According to CellProfiler 3.0 (“we’ve also made changes to CellProfiler’s underlying code to make it faster to run and easier to install, and we’ve added the ability to process images in the cloud and using neural networks“) it’s much faster now. I think it would be fair to compare run times using the latest version of CellProfiler. Please add those to the already existing run time comparison of Adipocyte U-Net vs Adiposoft and update corresponding text in the abstract if necessary.

We agree with the reviewer it would be ideal to compare our method with the latest published methods. This manuscript was first posted on Biorxiv in June 2019. When we were carrying out the analysis (2017-2018), CellProfiler 3.0 was not published. We furthermore believe that the manual exclusion of artefacts compared to runtime is the most time critical step in the CP workflow. Considering the manual revision of each image improvements in runtime of the same pipeline with a newer CP version would in our opinion almost be negligible for the total time required to obtain area estimates from an input image. Additionally and most importantly, the deep learning functionality of CellProfiler 3.0, which is comparable to our method in terms of algorithmic implementation, requires users to submit their data to a cloud service, rather than run locally. From CellProfiler 3.0’s abstract:
“.. and we’ve added the ability to process images in the cloud and using neural networks (deep learning).”

This is cumbersome and in many cases when using restricted-access clinical data, not a feasible option. Furthermore, we process hundreds of thousands of images for this work, and even for the purpose of benchmarking our method, it would be difficult to upload these to a cloud service. We therefore leave additional benchmarking for future work.

Please provide a brief description of how Adiposoft (p. 22), CellProfiler (description is missing) and floating adipocyte fractions (p.20) quantifications were generated (e.g. software parameters, were all adipocyte fractions after collagenase treatment used or only certain fractions, etc.). This can be very brief but it would be nice to be able to roughly follow how other methods were used.

We thank the reviewer for noticing this omission. Adiposoft requires no parameters, only the image in which you would like to analyse. For the floating adipocyte fraction work, we have now added a brief description of how fat cells were isolated, which fraction was used and how adipocyte size was determined. For CellProfiler we describe essential parameters of adipocyte size determination such as lower threshold and minimal number of counted cells. A detailed description of the method, from slide preparation to CellProfiler output, including a download link for the pipeline that was used can be found in the specified literature [(Berry et al. 2014)](https://paperpile.com/c/u6JZzU/vtcD). The Cell Profiler 2.0 pipeline developed by Berry et al. is also available in our github repository.

1) Collagenase digestion

The total adipocyte fraction was isolated as described earlier (Skurk et al. 2007). Briefly, the dissected adipose tissue was minced with scissors and digested in Krebs-Ringer phosphate (KRP) buffer containing 4 % BSA and 100 U/ml collagenase for one hour under moderate agitation. To isolate the floating total adipocyte fraction samples were filtered through nylon meshes (2000 µm and 250 µm pore size) and washed with KRP buffer three times. To estimate adipocyte size a small aliquot of the total adipocyte fraction was spread on a microscope slide and adipocyte diameter was determined using an integrated object micrometer by manually reading out the size of 100 cells.

2) Cell Profiler

Images from adipose tissue sections were obtained and size determination was carried out using a previously published CellProfiler 2.0 pipeline [(Berry et al. 2014)](https://paperpile.com/c/u6JZzU/vtcD). Identified objects < 314 µm2 ( = 20 µm diameter) were excluded as these commonly represent artefacts. Each output image was manually analyzed to exclude remaining artefacts and structures that were not identified correctly. At least 200 identified objects were used to calculate mean adipocyte sizes.

Is it known from the literature that adipocytes in subcutaneous depots are larger than their visceral counterparts or is this reported for the first time. Please specify.

Yes, this is fairly well known and we apologize for not providing citations to support this statement. We have now added the following citation: [https://www.ncbi.nlm.nih.gov/pmc/rticles/PMC4573191/](https://www.ncbi.nlm.nih.gov/pmc/articles/PMC4573191/)

Related to this question, can Adipocyte U-Net be applied to other tissues that are not predominantly fat tissue (e.g. arteries, muscles) but where biopsies are often “contaminated” or infiltrated with adipocytes. Do you expect adipocyte size differences in these different tissues?

This is an interesting question that we have considered. For example, GTEx provides histology imaging data on 40+ tissues. As stated, applying the adipocyte U-net to e.g., perivascular, epicardial and intermuscular adipose tissue could be of high interest as there is evidence for a role of ectopic fat in insulin resistance, atherosclerosis and cardiovascular disease [(Neeland et al. 2019). Other applications could be to measure bone marrow adipocyte size or](https://paperpile.com/c/u6JZzU/cizT) quantify lipid accumulation in non adipose cells such as hepatocytes during NAFL/NASH. We would expect the largest adipocyte size in the subcutaneous and visceral depot as these reflect the two major triglyceride storage sites. Adipocytes from other depots to our knowledge are usually smaller compared to their sc/vc counterparts as reported by [(Miggitsch et al. 2019; Chatterjee et al. 2013; Eiras et al. 2010)](https://paperpile.com/c/u6JZzU/OTKk+9Oun+h8BT).

It would be possible to apply our method to any histology imaging that contained adipocytes. However, it may be ideal to fine-tune our algorithm on a few additional images (10’s) of the reference tissue studied. This would be an interesting avenue of future work and touches on the issues of domain adaptation and/or transfer learning in the machine learning literature.

We have expanded the discussion to include this.

*“We foresee Adipocyte U-net or other deep learning based methodologies rapidly being redeployed and retrained using transfer learning so they can be applied to tissues other than adipose to detect adipocytes or further still, other cell types.”*

Often multiple biopsy samples are captured in the same image of a proband and some images show high inter-biopsy heterogeneity. Can Adipocyte U-Net be used to quantify the inter-biopsy heterogeneity to e.g. identify outlier images/proband or use it as an endophenotype itself?

Yes. This is a very interesting idea. As adipocyte U-net can quantify adipocytes in any histology image, it would be possible to calculate inter and intra subject variability. Indeed, aspects of this are captured in our adipocyte area variance GWAS.

Can the authors comment on the bimodal distribution seen in visceral tissues of Fig. 3? Did the authors check if this is a sampling issue or is there an underlying biological mechanism known? Providing example images of samples of each of the two peaks might be helpful.

Interestingly, we see bimodality in BMI distributions. For example in the MOBB cohort, we see a bi-modal distribution of lean controls and morbidly obese patients. However, this would not manifest as depot (visceral) - specific bimodality, so it still remains unclear why this is the case. Supplementary figure 9 plots two individuals at the extreme of the distribution (largest and smallest variable adipocytes). When we check the GTEx clinical metadata, the individual with small adipocytes whose cells look like they are undergoing hypertrophy, was diagnosed with anorexia - This bimodality may represent sampling, but our analysis is inconclusive.

Did the authors check if known BMI GWAS hits replicate in their GWAS data of mean (or variance) adipocyte area? Or vice versa, do the suggestive signals of their GWAS replicate in published, highly powered BMI GWAS studies? The comparison of adipocyte size and BMI GWAS results would give further insights how endophenotypes and clinical phenotypes relate to each other.

We thank the reviewer for this important question. We had not initially done this analysis - an oversight on our part - but have now done so and included a summary of the results in the manuscript.

In brief, we did exactly as the reviewer suggests, taking the index SNPs from large-scale genome-wide association studies of BMI and waist-hip-ratio adjusted for BMI (WHRadjBMI) and looking them up in our meta-analyses of histology phenotypes. Because we have previously performed sex-specific GWAS in BMI and WHRadjBMI, we also looked up index SNPs from those GWAS in the sex-specific meta-analyses of our histology phenotypes. As suggested, we also performed the reverse analysis, looking up suggestive signals from our histology meta-analyses in the BMI and WHRadjBMI GWAS. We have summarized the results in the results section.

In looking up the index SNPs from large-scale BMI and WHRadjBMI GWAS, we found that 4-7% of the index SNPs achieved a p-value < 0.05 in our histology GWAS, consistent with the null hypothesis. Indeed, if we perform an enrichment test on these nominally-replicating SNPs, none of the tests are significant after multiple test correction. Similarly, only 1 of the suggestive (p < 5e-7) signals, the SNP rs72811236, achieved a p-value < 0.05 in either the large-scale BMI or WHRadjBMI GWAS. Specifically, rs72811236 has a nominal p-value (p = 0.0048) in the sex-specific meta-analysis of BMI that analyzed women only. The lack of enrichment in association signal at known BMI and WHRadjBMI loci, as well as the limited number of suggestive SNPs in our histology GWAS with nominal signals in large-scale fat distribution GWAS, are results most likely attributable to the limited power in our histology GWAS. Even the ‘suggestive’ SNPs may be SNPs that, due to randomness, have strong p-values in our current analysis but would decrease in significance if more samples were available. The relatively strong association of rs72811236 in our histology GWAS and its nominal p-value in the women-specific BMI GWAS indicates a potential additional BMI locus that remains to be discovered. But here again, more samples will be necessary to establish whether or not this is indeed a bona fide locus with either a role in adipocyte morphology and/or BMI etiology.

Are the authors planning to make the individual-level mean and variance adipocyte size data available? I was not able to find them among the supplementary tables.

This data is **already** available on the Github repository (including all cohorts, adipocyte mean by cohort and adipocyte variances by cohort): <https://github.com/GlastonburyC/Adipocyte-U-net/tree/master/predictions_per_cohort>

Out of curiosity, besides adipocyte size can Adipocyte U-Net be used to estimate the number of adipocytes of the whole image and use it as a phenotype for association with traits and genotypes? Or quantify the overall area of an image that contains adipocyte e.g. to automate the quantification of adipose tissue contamination/infiltration in e.g. arteries, muscles, etc?

We thank the reviewer for the question. Yes, as Adipocyte U-net segments adipocytes, it obtains both a count and an area calculation per cell. Histology images are very large, usually more than 100,000 x 50,000 pixels. To make the problem tractable, we tile the image into 1024 x 1024 sections. It would be entirely possible to get a total count and total area of the image and use that as a quantitative phenotype downstream.

Probably out of scope for this paper but did the authors check if mean adipocyte size (or its variance) is correlated with in silico adipocyte estimates measured by the authors themselves (Glastonbury et al. AJHG 2019) or available through the gtex portal (<https://storage.googleapis.com/gtex_analysis_v8/interaction_qtl_data/GTEx_Analysis_v8_xCell_scores_7_celltypes.txt.gz>, Kim-Hellmuth et al., biorxiv 2019)?

We thank the reviewer for the question. We stopped analysing data for this manuscript before Kim-Hellmuth *et al*., biorxiv 2019 was published. The reviewer cites our previous work on *in-silico* cell type estimation from GTEx. CIBERSORT, which Glastonbury et al., 2019 used to obtain *in-silico* estimates, only calculates relative proportions of cells, it does not quantify absolute counts. For example, if one were to estimate macrophages and adipocytes with CIBERSORT, a sample may be predicted to be 0.05:0.95. Whilst if one in addition, estimated Neutrophils, the relative proportions would change (e.g., 0.01:0.15:0.84). We therefore thought it was inappropriate to compare relative *in-silico* measurements to absolute image derived measurements, as they aren’t capturing the same thing. However, in this manuscript we do compare against two state of the art and computational methods and the commonly used lab protocol for measuring adipocyte area. We believe three comparisons with leading methods is sufficient.

Minor points:

When reading the abstract for the first time I was not sure what is meant by “adipocyte area” as it can be the size of an adipocyte but it can also be the area of an image that is occupied with adipocytes (similar to % fat tissue). It might help to use “mean adipocyte size” at the beginning or explain in a few words what is meant with adipocyte area.

We thank the reviewer for the clarification. We have updated both the abstract and the introduction to use ‘mean adipocyte area’ as we believe this is indeed clearer.

Fig. 2: Can the authors add a diagonal line to better visualize that Adipocyte U-Net tends to estimate smaller adipocyte sizes. If the MOBB cohort was analyzed using the old CellProfiler it should be rerun using CellProfiler 3.0 to compare Adipocyte U-Net with the latest versions of other methods.

We thank the reviewer for their comment. As discussed above, CellProfiler 3.0 was not available when we were conducting the analysis, and as such could not include these analyses in our work. Further, we believe there are severe limitations in its usability when comparing many images.

P. 7: please provide a correlation plot that shows Adipocyte U-Net vs fat cell size from collagenase digestion.

We have added the required analysis to our manuscript (Supplementary figure 15)

P.

9: Last sentence says “…one of the four cohorts analysed (Supplementary Figure 7)”, specifying the panel, I think it is panel B, would be good.

We have updated the manuscript accordingly.

P. 12: please provide correlation plot of mean adipocyte area and sample ischemic time.

Whilst marginally significant, we don’t believe this plot adds anything to the manuscript in addition to the provided beta, as the correlation only explains 1.6% of the variance.

P. 20+21: text says “Using the remaining ~65,000 SNPs, we checked samples for inbreeding…” but the last paragraph of the MOBB data set says “The final cleaned dataset included 190 samples and ~700,000 SNPs.” Is this a typo? If not, please add more details to this method section to clarify which step removes how many SNPs.

Apologies to the reviewer for the confusion. Since sample QC is performed before SNP QC, those 65,000 high-quality SNPs are used strictly for sample QC and are defined using a stringent set of criteria (please see the Methods just above this for details). Using such a stringent set of SNPs prevents any sample QC steps from being potentially skewed by any low-quality SNPs in the data. Once sample QC is complete, we go back to the full genotyping dataset (i.e., all of the SNPs originally genotyped and a set far larger than the strictly-defined set of high-quality SNPs) and begin SNP QC. We have added detail on how many SNPs we begin with during SNP QC and how many we end with (as well as clarifying detail in the sample QC section) so that this section should hopefully clarify the QC process.

Please provide further statistics (boxplot, mean/median line, p-values of the group comparisons) mentioned in the text or legend for fig. S10, S11 and S13 for easier comparison. MOBB data was tested for the adipocyte area association with T2D but the corresponding plots are missing. Please add those as additional panels to fig. S11.

Supplementary Figure 10 is cited alongside Supplementary Figure 7A, which contains the most useful statistical information, namely standard errors, P-values and N for the meta-analyses between depots.

Fig. S14: The text says “when we repeat this analysis in GTEx visceral fat samples and ENDOX subcutaneous fat samples, we see no evidence of association” however only ENDOX samples are shown in fig. S14. Unless fig. S13A shows GTEx visceral fat samples please provide the corresponding GTEx plot.

The reviewer is indeed correct about Fig S13A and we have clarified this in the figure legend.

“rs1558902.chr16.53803574.cohort-level.gwas.results” and the other three supplementary tables contain cohort names such as “frayling”, “ndog” and “julius”. Please specify which name belongs to which cohort in a separate tab or in the header. Or adjust names to match the ones used in the manuscript (Endox, mob, fatdiva).

We thank the reviewer for catching this detail and have fixed the tables accordingly.

If the number of references is not limited I would suggest updating (or at least adding) the more recent citations (e.g. CellProfiler 3.0 McQuin et al., 2018 and GTEx most recent preprint (<https://www.biorxiv.org/content/10.1101/787903v1>).

We have added both references to the manuscript as they are both relevant and valuable contributions to the field.

**Have all data underlying the figures and results presented in the manuscript been provided?**

Large-scale datasets should be made available via a public repository as described in the *PLOS Computational Biology* [data availability policy](http://journals.plos.org/ploscompbiol/s/data-availability), and numerical data that underlies graphs or summary statistics should be provided in spreadsheet form as supporting information.

Reviewer #1: Yes

Reviewer #2: No: Installation instruction and user manual are not provided to the reviewer to test the software and reproduce results.

We think this is an unfair judgement. We have done something that is (sadly) quite rare in the scientific community, and that is to make all our data, code and models publicly available with no licence. However, we have tried to address the reviewers concerns by adding a more step by step guide, tutorial and interactive notebook.
