# Supplementary material for: Machine Learning based histology phenotyping to investigate the epidemiologic and genetic basis of adipocyte morphology and cardiometabolic traits
Source: PLoS Comput Biol. 2020 Aug 14;16(8):e1008044. doi: 10.1371/journal.pcbi.1008044 (PMC7449405; doi:10.1371/journal.pcbi.1008044)
Supplement: Attachment — Submitted filename: Glastonbury_25Mar20_response_26May.docx [file pcbi.1008044.s007.docx]

**Firstly, we would like to apologise to the reviewer for any misunderstanding and secondly thank them for their time for improving our work. We have addressed all of the reviewers points below, giving line changes and highlighting all new additions to the manuscript**

Glastonbury and colleagues have submitted a revised version of their manuscript.

In my original (Reviewer 1) review, I defined a number of author action items. The following were addressed:

Author action item:

- Please add a short discussion somewhere whether this general approach (genetic association studies with quantitative data from histological images is completely novel or has some precedent.

Addressed.

Author action item:

- Where it states “Given the different metabolic and physiological roles subcutaneous and visceral adipose tissue depots play…” a citation would be valuable.

Addressed.

Author action items:

- With genotyping data available, why was ancestry not assigned using genetic information? How variable were these subjects in terms of their ancestry?

I note that in the Methods section the following text is included (not sure whether this was in the original and I missed it, but it addresses the issue):

*“Six samples appeared to have some amount of non-European ancestral background, while the majority of samples appeared to be of European descent.”*

Author action item:

- The causality statement “While this suggests that BMI might act as the primary modifier of adipocyte size…” is probably too assertive, as adipocyte size is later described to mediate BMI: “mean adipocyte size in visceral fat accounting for, on average, 25% of the variance of BMI”. These statements are a departure from the usual, more neutral descriptions in terms of associations, in which the direction of causality is not assumed.

I accept the authors’ rebuttal.

However, the other issues I brought up were addressed only in the response to reviewers, and I see no indication that any of these was addressed in the text. Listing the three:

1. The rationale for the study is that increased fat underlies increased body mass index (BMI), and that increased fat is due to either the hyperplasia (number of cells) or hypertrophy (size of cells) of adipocytes. Of these, it is only really practical to measure size, as it would be enormously challenging to count adipocytes accurately.

Author action item:

- Please discuss whether anyone has ever tried to measure adipocyte number, or even how in theory this might be performed.

There are indeed published methods specifically designed to count adipocytes, for example AdipoCount (https://www.ncbi.nlm.nih.gov/pmc/articles/PMC5826178/).

Another common and published approach would be to calculate the total or depot specific adipocyte number from fat cell volume. This is best done in floating mature adipocytes from collagenase digestion as you always measure the maximum cell diameter (which is not always the case in histology). Assuming a density of adipose tissue of 0.9 and knowing the total fat mass by either weighing the fat pads (mice) or dexa/bia measurements in humans you can calculate the total number of fat cells in the body. This has for example been done by Spalding et al. (2008). However, we agree with Reviewer #1 that adipocyte surface area is a much more interesting measurement.

**We thank the reviewer again for their suggestion and apologise for our omission, we have now added the following amendments to the manuscript:**

Lines [88-90]

Whilst adipocyte counting software exists[^15^](https://paperpile.com/c/VCmO4E/1n1J), we chose to focus on adipocyte area as a more tractable problem to solve. However, as a by-product of measuring adipocytes, we also get an approximate count (proportional to total fat mass).

As cell counting isn’t the primary aim of our manuscript, and that all our data are derived from human histology, we did not include any further discussion about how one would go about measuring the number of adipocytes using DEXA, mice fat pad measurements etc (as detailed above in red).

2. The authors find no loci reaching significance for association with adipocyte size, but then focus on two loci believed to influence adipocyte size, *KLF14* and *FTO*, not finding any evidence to support their roles, but expressing certain valuable caveats in reporting their results.

Author action item:

- Please explain what a “female-specific type 2 diabetes-imprinted locus” is. Uncited and unclear to a reader.

We apologise if this was unclear. KLF14 exhibits imprinted expression from the maternal allele. The lead SNP at KLF14 is associated with T2D, but only in women, hence it is a female-specific diabetes locus. For more detail, please see the original KLF14 manuscript, which we cite (https://www.ncbi.nlm.nih.gov/pubmed/29632379).

**We agree with the reviewer and have therefore expanded the following section:**

*Lines [332-334]*

*KLF14* is a female-specific type 2 diabetes-imprinted locus, it is only expressed from the maternally inherited allele. Additionally,  rs4731702 has been linked to T2D risk in recent GWAS, with the locus only significant in female samples[^38^](https://paperpile.com/c/VCmO4E/OmZm).

3. The major value of this paper is not in terms of novel insights, but in showing a path to a valuable approach to understanding the intermediate cellular phenotypes in human diseases. The Adipocyte U-Net tool is obviously cell type-specific, but it has to be assumed that other deep learning-based methods can be developed for other cell types.

Author action item:

- Please comment on how relatively easy or difficult it will be to develop comparable approaches that work for cells other than adipocytes. Are adipocytes relatively easy, will any cell types be predicted to be difficult, what sort of approaches would be need to count cell subtype proportions within a tissue or image area.

Whilst it would take some work to adapt this algorithm to work with other cell types, the algorithm is data efficient and using the principles of transfer learning, it could potentially be fine-tuned on fewer than 50 images of a new cell type. Adipocytes represent a rather easy case as Adipose tissue is quite homogenous. Complicated mixtures, such as the placenta (https://arxiv.org/abs/1804.03270) are more difficult, but it is still possible to estimate different populations of cell types present in the tissue histology.

**This is a really interesting point and again, an omission on our part not to include it in the revised manuscript. We have added this to our discussion (see below):**

Lines [431-437]

Adipose tissue represents quite a homogenous and therefore easy tissue for segmentation, with many other tissues, such as the placenta, being a much more complicated mix of cells and structures [(Ferlaino et al. 2018)](https://paperpile.com/c/VCmO4E/JEMP). Adipocyte U-net could be rapidly adapted to other cell types using as few as 50 annotations and the principles of transfer learning [^45^](https://paperpile.com/c/VCmO4E/R6T6). We envisaged future work to take advantage of the recent successes of meta-learning, in which many similar tasks, such as classifying or segmenting cells across a range of image types, are solved at the same time and any future task can be adapted to with very few gradient updates.[^46^](https://paperpile.com/c/VCmO4E/4jnD)

These were not brought up so that I could have a discussion with the authors, they were raised as issues that needed to be addressed for readers of the manuscript.

Please revise the manuscript (or point me to where I missed the appropriate revisions) and then I undertake to re-review these changes quickly. The responses provided above are fine – but they need to be in the paper!

**We appreciate the reviewers help with this and have therefore aimed to amend the manuscript as appropriate and to respond to these items ASAP (within a week of receipt).**
